# 2748. Ceftolozane/tazobactam For the Treatment of Cystic Fibrosis Patients: Results from a French Prospective Cohort Study

**DOI:** 10.1093/ofid/ofad500.2359

**Published:** 2023-11-27

**Authors:** Xavier Bourge, Brune Akrich, David Boutoille, Isabelle Brassac, Carole Mackosso, Linsay Monteiro Tavares, Joy Mootien, Fabrice Ruiz, Jean-François Timsit, Bernard Castan, Pierre-Regis Burgel

**Affiliations:** MSD France, VOURLES, Rhone-Alpes, France; Merck Research Labs, MSD, Puteaux, Ile-de- France, France; CHU de Nantes, Nantes, Pays de la Loire, France; MSD France, VOURLES, Rhone-Alpes, France; MSD France, VOURLES, Rhone-Alpes, France; Clinsearch, Malakoff, Ile-de- France, France; GHRMSA Hopital Emile Muller, Mulhouse, Alsace, France; Clinsearch, Malakoff, Ile-de- France, France; HU PARIS NORD SITE BICHAT APHP, Paris, Ile-de- France, France; Centre Hospitalier de Périgueux, Périgueux, Aquitaine, France; Hopital Cochin, PARIS, Ile-de- France, France

## Abstract

**Background:**

The aim of this prospective multicentre, French observational study was to describe the conditions of Ceftolozane/Tazobactam (C/T) use in hospital settings, and outcomes. This sub-analysis focuses on the cystic fibrosis (CF) patients included in the overall cohort.

**Methods:**

Adult patients suffering from CF having received at least one dose of C/T and followed up as per routine clinical practice until stop of C/T were included in this analysis. Additional data related to CF were collected.

**Results:**

Between October 2018 and December 2019, 63 patients with CF were enrolled from 28 sites. Mean age was 33.6 years and 44.4% were males. 12 patients (19.0%) received a lung transplant, 46.0% had comorbidities with diabetes (34.9%) being the most frequent. Most patients (69.8%) had normal renal function, almost one-third (28.6%) were immunosuppressed. One-half of patients presented with class 2 mutation of Cystic Fibrosis Transmembrane Conductance Regulator (CFTR) gene. None of the patients received CFTR modulators due to non-availability during the study period.

About one-half of patients (52.4%) experienced intolerance/allergy to antibiotics; of these, 78.1% was associated with Ceftazidime.

Microbiology results showed that *Pseudomonas aeruginosa* was the most predominant pathogen representing 89.9% (71/79) of the isolates. C/T showed a very high susceptibility rate of 91.5% across those strains (Table 1). C/T demonstrated higher susceptibility rates than other B-lactam agents (Table 2).

Treatment duration until complete cure had a median of 15 days and occurred in 44.4% of patients. Treatment duration until partial cure or end of treatment was a median of 15 days and occurred in 46% of patients (Table 3). Only 2 patients experienced an adverse event leading to a discontinuation of treatment.

Treatment with C/T had a positive impact on pulmonary function measured by FEV1 (Table 4) with a mean increase after treatment from 1.33L (range 0.5 - 4.1L) to 1.47L (range 0.6 - 4.6L).

**Figure:**
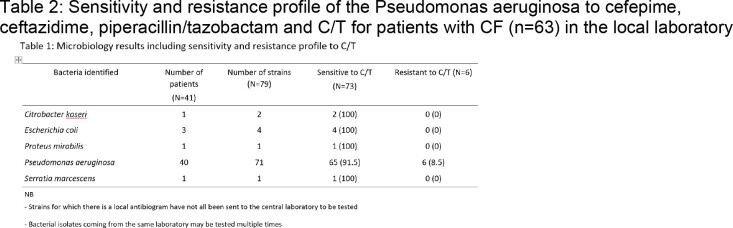

**Figure:**
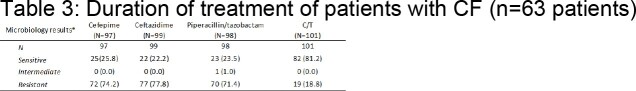

**Figure:**
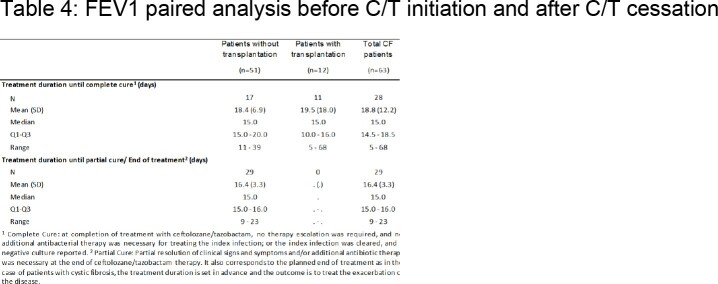

**Conclusion:**

These results suggest that C/T is an effective and safe option for the treatment of CF patients with a potential positive impact on FEV1 when treating bacterial infections mainly caused by *Pseudomonas aeruginosa*.

**Figure:**
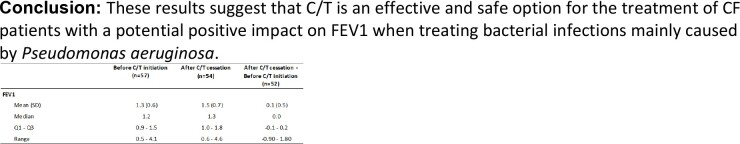

**Disclosures:**

**Xavier Bourge, PharmD**, Merck: Employe **Brune Akrich, MD**, MSD: Employee **David Boutoille**, MSD France: Board Member **Isabelle Brassac, Scientist**, MSD France: salary **Carole Mackosso, n/a**, MSD France: MSD employee **Jean-François Timsit, MD**, merck: Advisor/Consultant **Bernard Castan, MD**, ADVANZ: SPEAKER MODERATOR|BIOMERIEUX: SPEAKER MODERATOR|GILEAD: Advisor/Consultant|MSD France: Board Member|MSD France: SPEAKER MODERATOR|SANOFI: Advisor/Consultant|SHIONOGI: Advisor/Consultant **Pierre-Regis Burgel, MD,PHD**, ASTRA-ZENECA: Honoraria|BOEHRINGER INGELHEIM: Honoraria|CHIESI: Honoraria|GSK: Honoraria|INSMED: Honoraria|NOVARTIS: Honoraria|PFIZER: Honoraria|VERTEX: Grant/Research Support|ZAMBON: Honoraria

